# Analysis of the causes of cervical lymphadenopathy using fine-needle aspiration cytology combining cell block in Chinese patients with and without HIV infection

**DOI:** 10.1186/s12879-020-4951-x

**Published:** 2020-03-14

**Authors:** Lei Sun, Liang Zhang, Kun Yang, Xiang-mei Chen, Jia-min Chen, Jiang Xiao, Hong-xin Zhao, Zhi-yuan Ma, Li-ming Qi, Peng Wang

**Affiliations:** 1 Department of Pathology, Beijing Ditan Hospital,Captial Medical University, No. 8 Jing Shun East Street, Chaoyang District, Beijing, 100015 People’s Republic of China; 2 Center for Infectious Diseases, Beijing Ditan Hospital,Captial Medical University, No. 8 Jing Shun East Street, Chaoyang District, Beijing, 100015 China

**Keywords:** Cervical lymphadenopathy, Fine-needle aspiration cytology, Cell-block, HIV infection

## Abstract

**Background:**

Cervical lymphadenopathy refers to a frequently observed clinical presentation in numerous pathological conditions. A wide spectrum of diseases can cause cervical lymphadenopathy, irrespective of the fact that the patients are infected with HIV or not. The present study focuses on validating whether the causes of cervical lymphadenopathy differ significantly in HIV and non-HIV patients by using fine-needle aspiration cytology (FNAC) combining cell block.

**Methods:**

A total of 589 patients with cervical lymphadenopathy were recruited in the FNA clinic. The samples were obtained by an auto-vacuumed syringe that benefited the sampling more materials. The cytological smears were prepared by Hematoxylin and Eosin (HE), Periodic Acid Schiff (PAS), Gomori’s methenamine silver (GMS) and acid-fast staining. Cell blocks were made if required, and immunohistochemistry stain was performed on the cell block section.

**Results:**

The study found 453 (76.9%) patients with HIV and 136 (23.1%) patients without HIV infection. The average age of HIV-infected patients was 34.8 ± 10.2 years, which was significantly lower than that of non-HIV-infected patients (42.9 ± 18.1 years) (*p* < 0.01). Of all patients infected with HIV, 390 (86.1%) were males. This proportion was significantly higher than that of non-HIV-infected patients [65/136 (47.8%)] (*p* < 0.01). The major causes of cervical lymphadenopathy in HIV positive patients were mycobacterial infection (38.4%), reactive hyperplasia (28.9%), non-specific inflammation (19.9%), and malignant lesions (4.2%). In contrast, the most common causes in HIV negative patients were reactive hyperplasia (37.5%), malignancy (20.6%), non-specific inflammation (19.1%) and mycobacterial infection (12.5%). Opportunistic infections such as non-tuberculous mycobacteria (4.2%), cryptococcosis (1.5%), *Talaromyces marneffei* (1.5%) and other fungi (0.4%) were found only in HIV-infected individuals. Non-Hodgkin’s lymphoma (2.4%) was the most common malignant lesion in patients with HIV infection, followed by Kaposi’s sarcoma (0.9%) and metastatic squamous cell carcinomas (0.7%). However, the most common malignancy in non-HIV-infected patients was metastatic carcinomas (14%) including small cell carcinomas, adenocarcinomas, squamous cell carcinomas and hepatocellular carcinoma, which were noticeably greater than the HIV patients (*p* < 0.01).

**Conclusions:**

There were significantly different causes of cervical lymphadenopathy in HIV infected and non-HIV infected patients. FNAC was a useful diagnostic method for differential diagnosis of cervical lymphadenopathy.

## Background

Lymphadenopathy is a relatively common clinical presentation, especially in HIV infected patients. Cervical lymph nodes are most commonly involved among various lymphadenopathies. The presentation often changes from normal inflammation to a malignant state, sometimes being non-specific in nature.

Fine Needle Aspiration Cytology (FNAC) has been adopted as the primary diagnostic procedure for breast, thyroid gland, skin, and superficial lumps as well as enlarged lymph nodes. FNAC is an excellent diagnostic tool for HIV-positive patients with lymphadenopathy. It is a routine examination that is cheap, quick, safe, free of complications, well tolerated by the patient, and highly accurate [1]. The smear can be evaluated immediately and the procedure can be repeated several times to obtain further materials for diagnosis or special stains. Moreover, the residual components from cytological smears can be processed into cell blocks, which could provide the morphology and partial histological structures, and can be sectioned for immunohistochemical (IHC) staining.

Cytological evaluation and diagnosis of lymphadenopathy play an important role in HIV infected patients. This study, therefore, aimed to ascertain the utility of FNAC in evaluating enlarged cervical lymph nodes and to categorize the causes of cervical lymphadenopathy diagnosed by FNAC in patients with and without HIV infection at the hospital.

## Methods

Five hundred and eighty-nine cervical lymphadenopathy patients admitted to the Department of Pathology, Beijing Ditan Hospital, Capital Medical University in Beijing were recruited between June 2009 and February 2019. Written informed consent was obtained from all the patients and they were briefly explained about the FNAC technique before it was performed on them. The demographic details of the patients were obtained from the laboratory requisition forms submitted by clinicians as well as from the pathology reports. The study protocol was approved by Ethics Committee of Beijing Ditan Hospital, Capital Medical University.

FNAC was performed without anesthesia, using an auto-vacuumed syringe, bearing a latch at the bottom of the tube and a slot in the plunger, to be used in a pencil-grip manner. By employing this method a large amount of the sample can be collected. We flushed the aspirated material on slides and then spread it to make thin smears. The slides were fixed immediately with 95% ethyl alcohol and were stained using hematoxylin and eosin (HE), Gomori’s methenamine silver (GMS), periodic acid Schiff (PAS), and acid-fast staining for cytological examination. The remaining materials were processed into cell blocks, if required, via ethanol coagulation and formaldehyde fixation. All cell blocks were treated in the same way as surgical biopsy specimens, including formalin fixation, paraffin embedding, and sectioning at 4–5 μm thickness, followed by HE staining and immunohistochemical staining. Immunohistochemical analyses were performed by the avidin-biotin-peroxidase method with a Leica autostainer system on cell block sections.

The data obtained were analyzed using the SPSS (IBM statistics, Version 20.0, SPSS, Chicago, USA) statistical software. The differences between HIV positive and negative patients were obtained using independent Student’s t-test. Categorical variables were analyzed using the χ^2^ test. A *p*-value of < 0.05 was considered to be statistically significant. Continuous data have been denoted as mean ± SD.

## Results

Overall, the study comprised 453 (76.9%) patients with HIV and 136 (23.1%) patients without HIV infection. The proportion of male patients (390,86.1%) in the HIV-infected group was significantly higher than the number of male patients in the non-HIV-infected group (65/136,47.8%) (*p* < 0.001). The age of HIV positive patients ranged between 6 and 66 years, with the highest incidence (166/453,36.6%) in the age range of 21 to 30 years, followed by those in the range of 31 to 40 years (133/453,29.4%); this number was significantly higher than that of HIV negative patients (*p* = 0.001 and 0.004, respectively). The age range of HIV negative patients varied from 10 to 90 years, with the highest incidence (29/136, 21.3%) in the age range of 21 to 30 years. Yet, the incidences in age range of 61 to 70 years (21/136,15.4%) and above 70 years (9/136,6.6%) were significantly higher than that of HIV positive patients (p<0.001,both). The average age of HIV-infected patients was 34.8 ± 10.2 years, which was significantly lower than that of non-HIV-infected patients (42.9 ± 18.1 years) (*p* < 0.001) (Table 1). The average age of onset of lymphoma and metastatic carcinoma in HIV positive patients was also significantly lower than that in HIV negative patients (*p* = 0.003 and 0.049, respectively).

**Table 1:** Comparison of demographic profile between patients with and without HIV infection

	HIV positive patients	HIV negative patients	Total	*P* value
Sex				
Male	390(86.1%)	65(47.8%)	455	0.000
Female	63(13.9%)	71(52.2%)	74	
Age(y)				
Average	34.8 ± 10.2	42.9 ± 18.1	36.7 ± 12.9	0.000
Range	6–66	10–90	6–90	–
0–10	2(0.4%)	1(0.7%)	3	0.673
11–20	18(4.0%)	13(9.6%)	31	0.011
21–30	166(36.6%)	29(21.3%)	195	0.001
31–40	133(29.4%)	23(16.9%)	156	0.004
41–50	94(20.8%)	23(16.9%)	97	0.325
51–60	35(7.7%)	17(12.5%)	52	0.085
61–70	5(1.1%)	21(15.4%)	26	0.000
>70	0	9(6.6%)	9	0.000
Total	453	136	589	–

**Fig. 1:**
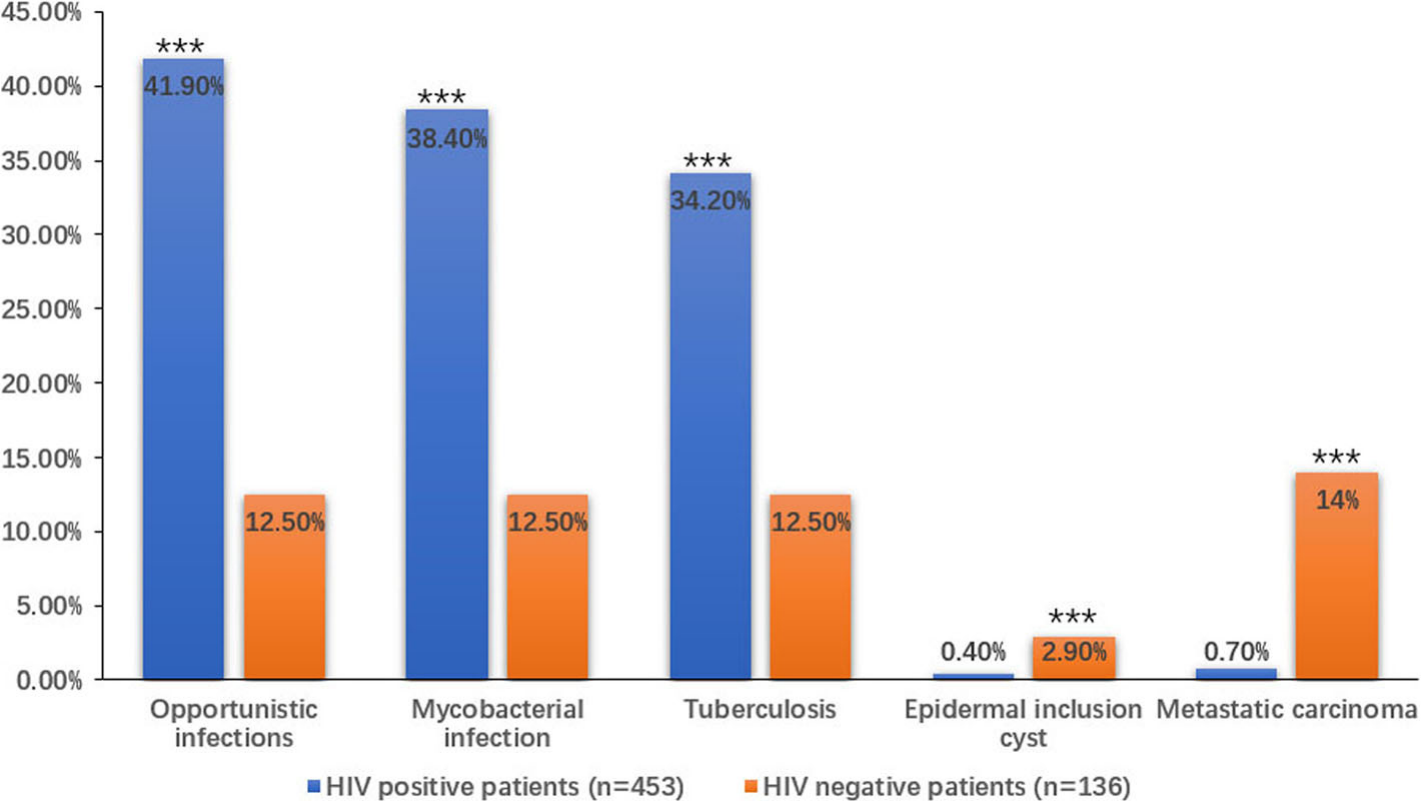
The positive rate of Opportunistic infections including Mycobacterial infection that are mainly tuberculosis in HIV/AIDS patients is significantly higher, and the positive rate of Epidermal inclusion cyst and Metastatic carcinoma is markly lower than that in HIV negative patients

Mycobacterial lymphadenitis (38.4%) was the most commonly observed lesion in HIV positive patients, followed by reactive lymphoid hyperplasia (28.9%), non-specific inflammation (19.9%), and malignant lesions (4.2%). In contrast, the most common causes in HIV negative patients were reactive hyperplasia (37.5%), malignancy (20.6%), non specific inflammation (19.1%) and mycobacterial infection (12.5%) (Table 2). The positive rate of Opportunistic infections including Mycobacterial infection that are mainly tuberculosis in HIV/AIDS patients is significantly higher than that in HIV negative patients(p<0.001,both), but the positive rate of Epidermal inclusion cyst and Metastatic carcinoma is markly lower than that in HIV negative patients(*p* = 0.008, <0.001, respectively) (Fig. 1).

**Table 2:** Comparison of cytological diagnosis of cervical lymphadenopathy between patients with and without HIV infection

Diagnosis	HIV positive patients(cases)	HIV negative patients(cases)	Total	P value	P value ^*a*^
**Benign lesions**					
Reactive lymphoid hyperplasia	131(28.9%)	51(37.5%)	182	0.058	0.232
Non-specific inflammation	90(19.9%)	26(19.1%)	116	0.847	0.397
Suppurative lymphadenitis	11(2.4%)	8(5.9%)	19	0.046	0.282
Granulomatous lymphadenitis	5(1.1%)	0	5	0.219	0.995
Kikuchi disease	1(0.2%)	1(0.7%)	2	0.364	0.726
Opportunistic infections	190(41.9%)	17(12.5%)	207	0.000	< 0.001
Mycobacterial infection	174(38.4%)	17(12.5%)	191	0.000	< 0.001
Tuberculosis	155(34.2%)	17(12.5%)	172	0.000	< 0.001
Nontuberculous mycobacteria	19(4.2%)	0(4.2%)	19	0.015	0.996
Cryptococcosis	7(1.5%)	0	7	0.145	
Talaromyces marneffei	7(1.5%)	0	7	0.145	
Other fungus	2(0.4%)	0	2	0.438	
Epidermal inclusion cyst	2(0.4%)	4(2.9%)	6	0.011	0.008
Lipoma	1(0.2%)	0	1	0.583	0.994
Vascular lesions	0	1(0.7%)	1	0.068	0.993
**Malignant lesions**					
lymphoma	12(2.6%)	9(6.6%)	21	0.029	0.296
Hodgkin lymphoma	1(0.2%)	0	1	0.583	
Non Hodgkin lymphoma	11(2.4%)	9(6.6%)	20	0.018	0.223
Kaposi’s sarcoma	4(0.9%)	0	4	0.272	0.996
Metastatic carcinoma	3(0.7%)	19(14%)	22	0.000	< 0.001
squamous cell carcinoma	3(0.7%)	12(8.8%)	15	0.000	0.003
adenocarcinoma	0	3(2.2%)	3	0.002	0.993
small cell carcinoma	0	3(2.2%)	3	0.002	0.993
hepatocellular carcinoma	0	1(0.7%)	1	0.068	
**Unsatisfactory aspirates**	3(0.7%)	0	3	0.341	0.996
Total	453	136	589	–	

^*a*^***P*-value adjusted for age and sex by logistic regression**

**Fig. 2:**
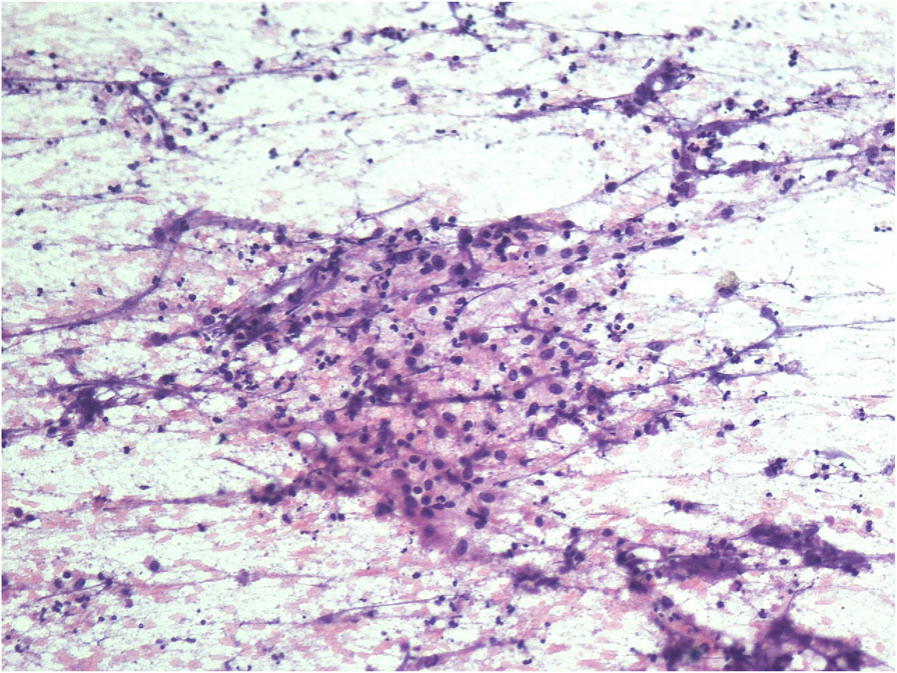
Epithelioid cells with caseous necrosis, neutrophilic aggregates could be observed HE × 200

**Fig. 3:**
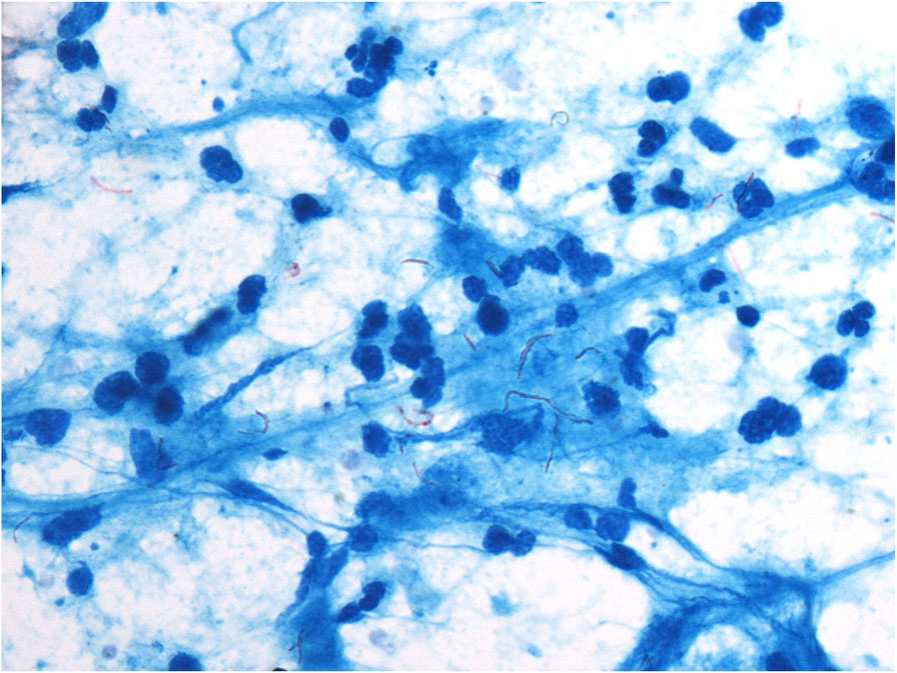
Tuberculosis were red, rod-shaped, slender and slightly curved Acid fast staining × 1000

Among the 174 HIV/AIDS patients with mycobacterial lymphadenitis, 155 cases were tuberculosis infections and 19 cases were non-tuberculous mycobacterial infections. The most common cytomorphological type in tuberculous lymphadenitis is caseous necrosis with epithelioid cell granuloma and multinucleated giant cells. In many cases, necrotizing suppurative inflammation and neutrophilic aggregates were observed (Fig. 2). Acid-fast staining of *Mycobacterium tuberculosis* confirmed the diagnosis when inflammatory exudates appeared, showing red, rod-shaped, slender and slightly curved mycobacteria (Fig. 3). In 122 out of 155 (78.7%) HIV positive patients and 8 out 17 (47.1%) HIV negative patients, the smears stained were positive for acid-fast stains. The rate of positive results in HIV/AIDS patients is significantly higher than that in HIV negative patients (*p* = 0.004).

**Fig. 4:**
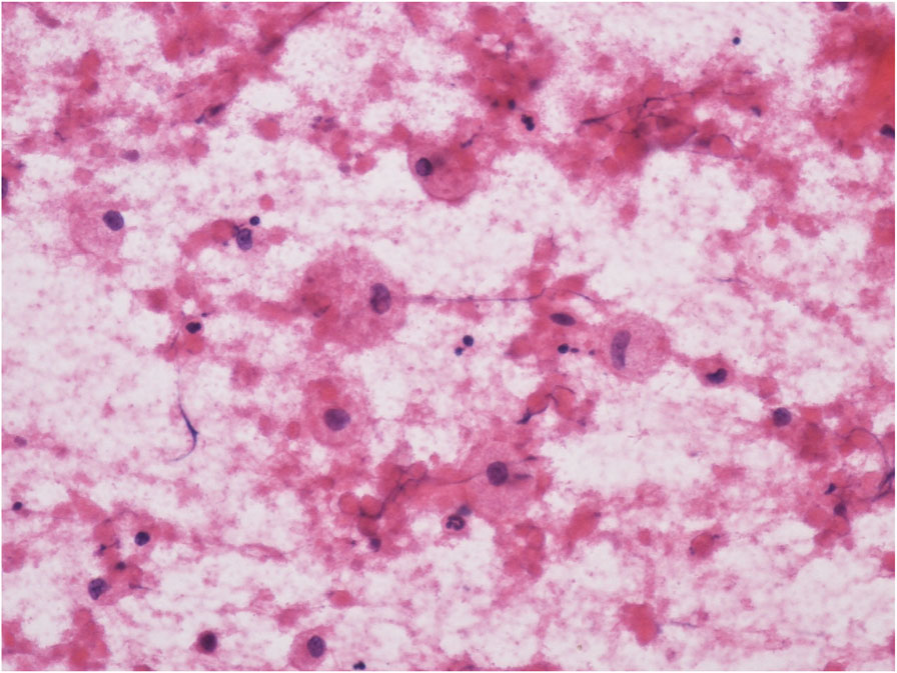
Smaears of MAC showed many macrophages with foamy cytoplasm HEx400

**Fig. 5:**
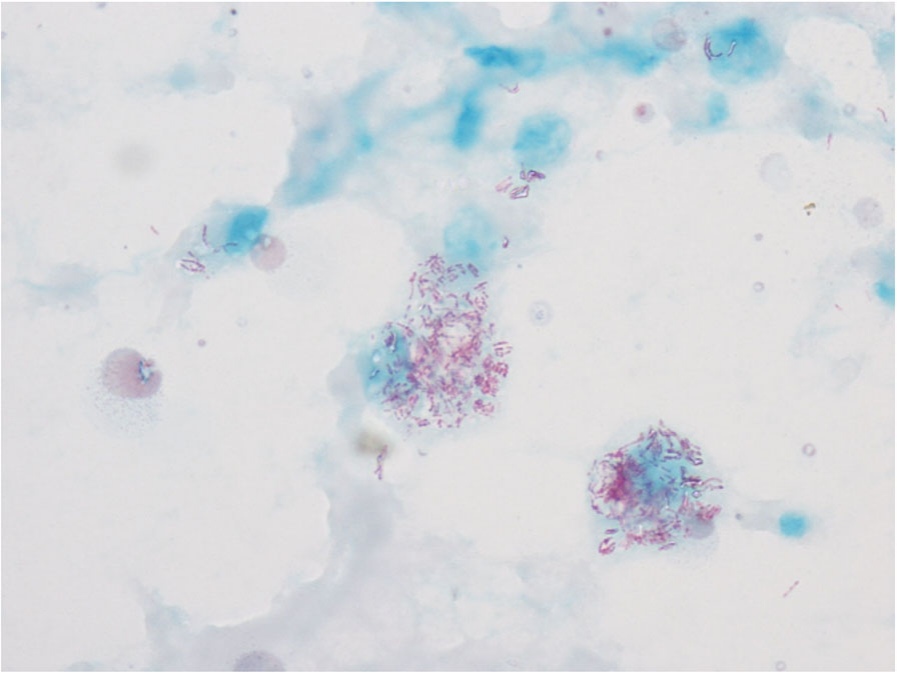
MAC mainly located in the cytoplasm of these macrophages Acid fast staing × 1000.

Nineteen cases (4.2%) were diagnosed with a non-tuberculous mycobacterial infection in HIV positive patients, which mainly comprised the *Mycobacterium avium* Complex (MAC). HE staining of smears showed many macrophages with foamy cytoplasm (Fig. 4); MAC was mainly located in the cytoplasm of these macrophages, which could also be shown by acid-fast staining (Fig. 5).

Reactive lymphoid hyperplasia was found to be the most frequent diagnosis in HIV negative patients (51/136, 37.5%); the smears displayed a polymorphous small and large lymphocytic population along with a few tingible body macrophages, monocytoid cells, dendritic reticulum cells, and plasma cells. All special stains gave negative results.

**Fig. 6:**
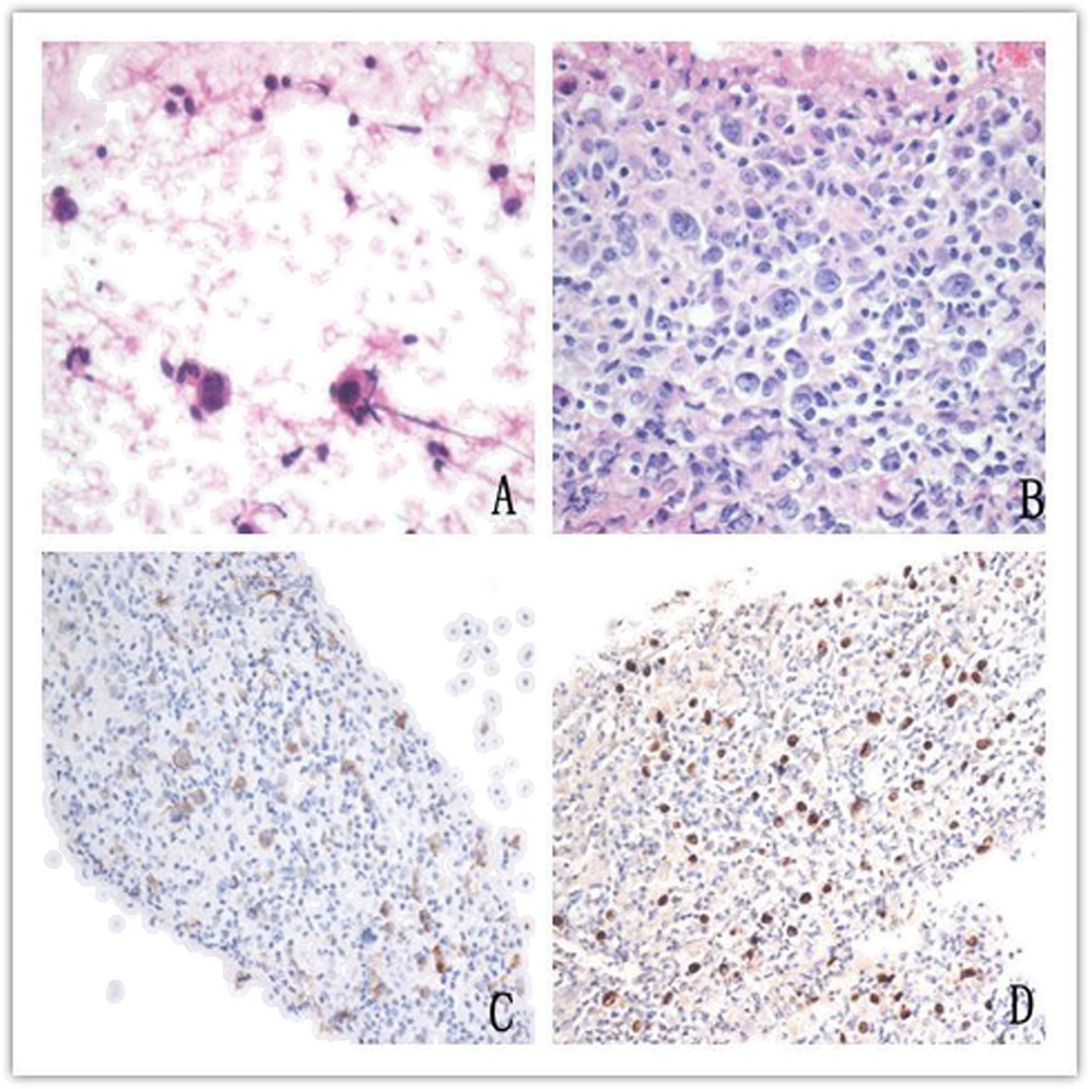
One case of Hodgkin lymphoma. **a**. cytological smear showed Reed-Sternberg cells and their mutant derivatives with small lymphocytes background. HEx400. **b**. Sections from cell blocks demonstrated many Reed-Sternberg cells. HEx400. **c**. Immunohistochemistry of cell block sections demonstrated CD30 positivity. × 200. **d**. In situ hybridization of cell block sections demonstrated EBV positivity. × 200

Malignant lesions mainly included lymphoma and metastatic carcinoma. Kaposi’s sarcoma was diagnosed only in four HIV positive patients. The remnant materials from cases in which tumor cells were found on the smears were processed into cell blocks, followed by immunohistochemical staining (Fig. 6). There were one (0.2%) Hodgkin’s lymphoma and eleven (2.4%) non-Hodgkin’s lymphoma cases among HIV positive patients, in contrast to nine (6.6%) non-Hodgkin’s lymphoma cases among HIV negative patients. The incidence of lymphoma in HIV negative patients was remarkably greater than that in HIV positive patients (*p* = 0.029).

**Fig. 7:**
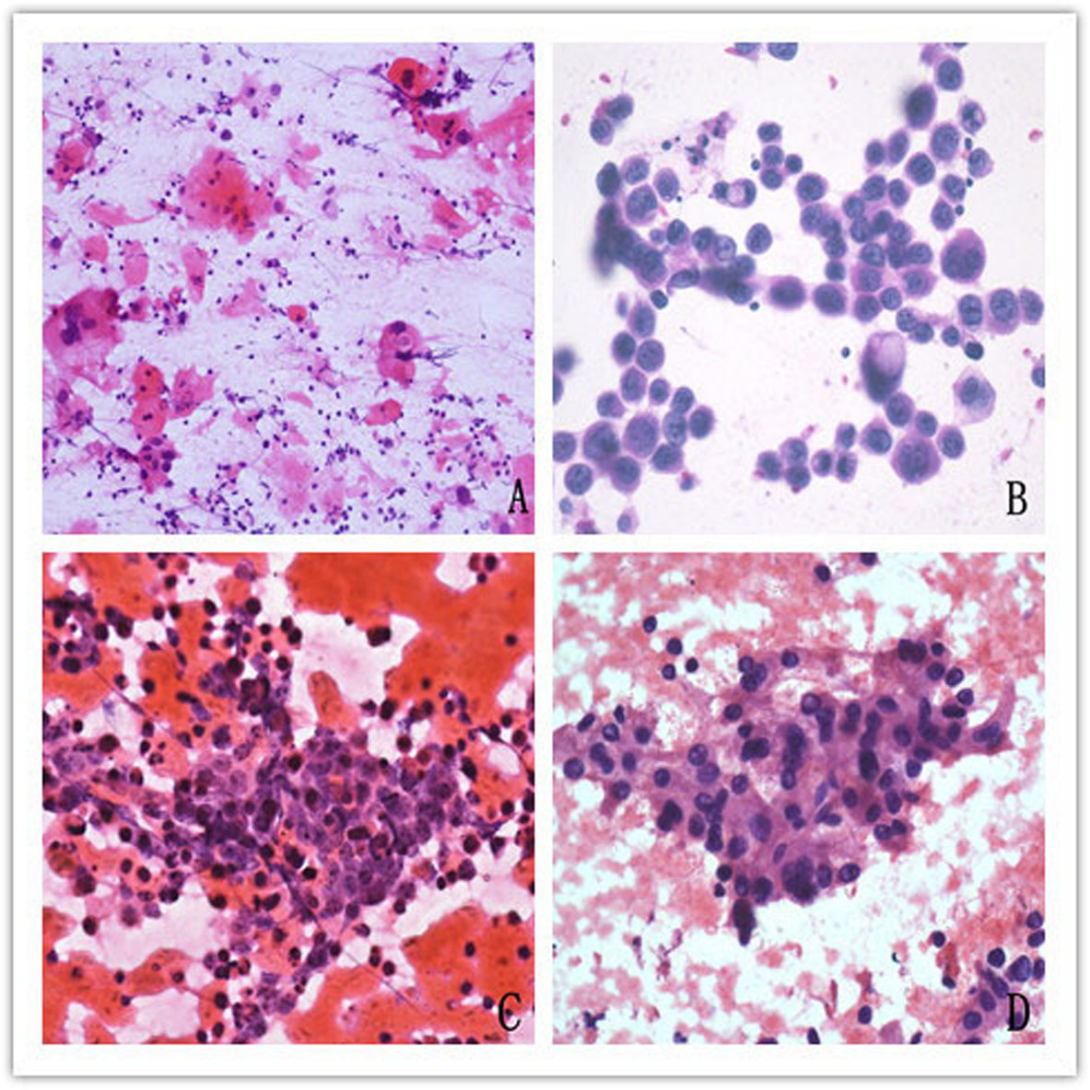
Metastatic carcinoma. **a**. Metastatic squamous cell carcinoma demonstrated spindly keratinized cells, intercellular bridges, dense cytoplasm and dirty necrotic background. HEx400. **b**. Cytological features of metastatic adenocarcinoma were glandular pattern, cells with vesicular nucleus, prominent nucleoli, intracytoplasmic mucin vacuoles. HEx400. **c**. Smear of metastatic small cell carcinoma showed nuclear molding, streaking of nuclear material. HEx400. **d**. Smear of metastatic hepatocellular carcinoma showed large prominent central nucleoli and rich eosinophilic cytoplasm. HEx400

Out of the 19 cases with metastatic carcinoma in HIV negative patients, the most common malignancy was metastatic squamous cell carcinoma (12 cases), while metastatic adenocarcinoma (3 cases), small cell carcinoma (3 cases) and hepatocellular carcinoma (1 case) ranked behind. There were only 3 cases with metastatic squamous cell carcinoma in HIV positive cases, in whom the incidence of metastatic carcinoma was markedly lower than that in HIV negative cases (*p* < 0.001). The cytological features that helped in the diagnosis of metastatic squamous cell carcinoma included the spindly keratinized cells, intercellular bridges, dense cytoplasm, and dirty necrotic background. Non-keratinizing squamous cell carcinoma exhibited cells with round to oval nuclei having coarse granular chromatin and prominent nucleoli. In metastatic adenocarcinoma, the characteristic cytological features included glandular pattern, cells with vesicular nuclei, prominent nucleoli and intracytoplasmic mucin vacuoles. Small cell carcinoma was characterized by nuclear molding and streaking of nuclear material that helped in its diagnosis. Large prominent central nucleoli and rich eosinophilic cytoplasm were frequently seen in hepatocellular carcinoma (Fig. 7).

**Fig. 8:**
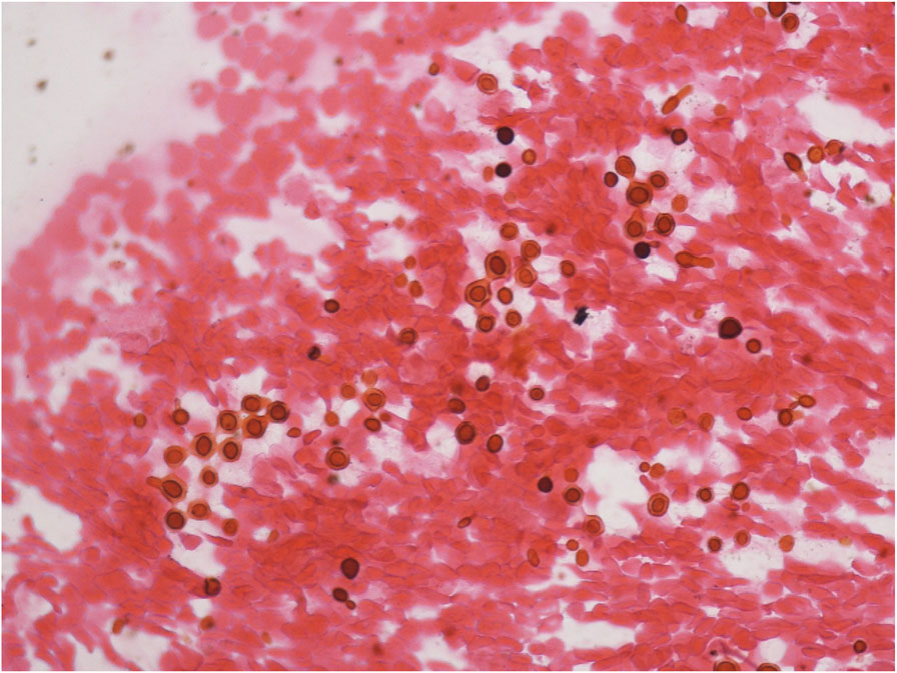
Cryptococcosis were the halo-like, narrow-necked budding yeast cells surrounded by a well-defined mucopolysaccharide capsule with hemorrhagic background. GMSx400

**Fig. 9:**
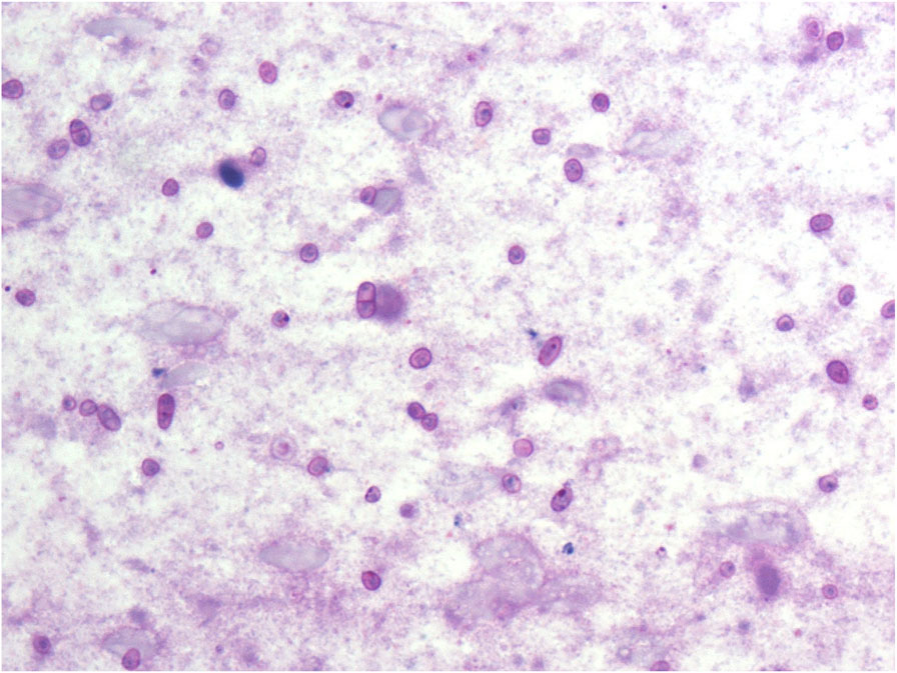
Talaromyces marneffei were the round or oval shape,some like a “sausage”, with a central dot-like structure, a prominent central septum was seen in some organisms. PASx1000

Opportunistic infections such as *Cryptococcosis* (1.5%), *Talaromyces marneffei* (1.5%), and other fungi (0.4%) were found only in HIV infected patients. The cytological diagnosis of cryptococcosis was based on halo-like, narrow-necked budding yeast cells surrounded by a well-defined mucopolysaccharide capsule, which stained positive for GMS and PAS (Fig. 8), and was accompanied by a foamy macrophage-rich inflammatory or hemorrhagic background. Smears of *Talaromyces marneffei* were characterized by round or oval shape, some like a “sausage”, bearing a central dot-like structure. These yeast-like organisms had a prominent central septum that was clearly evident with GMS and PAS staining (Fig. 9).

## Discussion

Lymph nodes refer to the most extensively distributed and readily accessible lymphoid tissue components, which are commonly affected in patients with HIV/AIDS. Lymphadenopathy is one of the most common presentations among HIV positive patients; it may be a presenting feature in about 35% of all the HIV/AIDS patients [2].

The neck comprises about 2/3rd of all lymph nodes of the body. Enlargement of the involved lymph nodes is relatively noticeable [3]. Cervical lymphadenopathy is a frequent presentation of numerous pathological processes, especially AIDS. The differential diagnosis of cervical lymphadenopathy includes many benign and malignant diseases. Since cervical lymphadenopathy is a commonly encountered clinical condition, a prompt and accurate diagnostic procedure must be carried out so that a proper treatment protocol can be initiated at the earliest. Surgical biopsy is the gold standard for its diagnosis. However, it often requires hospitalization, and is costly, time-consuming and not always free of complications. Fine needle aspiration cytology (FNAC) offers a good alternative. In the past decade, FNAC has been widely employed as an important diagnostic tool for evaluating cervical lymphadenopathy among HIV positive patients as it is less invasive, simple, quick and less expensive than excision biopsy [4]. The smear evaluation yields immediate results and the procedure can be repeated many times so as to obtain more materials, if required, for the diagnosis which is often done in conjunction with special stains, cell block, and IHC.

An optimized approach for FNAC and cell block preparation was adopted in the hospital. The critical modification was the use of an auto-vacuumed syringe, benefiting the sampling a larger amount of materials to make cell block. The aspirated material was flushed onto the slides and spread to obtain smears. The remaining materials were processed into cell blocks, which are composed of random cells and tissue fragments for visualizing tissue architecture [5]. As in the case of Hodgkin lymphoma described hereby, with cell block section, the morphological features of cell block are similar to histopathology. Cell blocks not only provide morphology and partial histological structures but can also be sectioned for IHC staining, thus allowing immunohistological evaluation. Therefore, patients with cervical lymphadenopathy must undergo FNAC and cell block, if required, for the diagnosis and accordingly, the next step of the therapeutic approach can be planned.

The HIV positive patient group comprised more males than females, which is consistent with other studies [6, 7]. However, in HIV negative patients, males were very few in number; the proportion was significantly lower than that in HIV-infected patients, thus indicating that HIV infection mainly affects males. The average age of HIV-infected patients was noticeably lower than that of non-HIV-infected patients. Age of HIV positive patients ranged from 6 to 66 years, with the highest incidence in the age range 21 to 30 years, followed by range 31 to 40 years; this result is similar to that of the research by Vanisri [8] and Shenoy [9]. In the study by Vanisri, the most affected age group was 21–30 years, while 31–40 years ranked behind in HIV incidence. In the study by Shenoy, the age group of 25–30 years was the most affected one, indicating that young people are at higher risk of HIV infection, it is, therefore, necessary to increase the efforts of disease prevention and control in youth.

Cervical lymphadenopathy is one presentation that results from various etiologies, having a wide spectrum from infectious to neoplastic conditions. In HIV/AIDS patients, HIV infection itself might produce lymphadenopathy, and various opportunistic infections and malignancies may also be responsible for the cervical lymphadenopathy encountered. The etiologies vary with nation and area. In under-developed countries, tuberculous lymphadenitis is a relatively common cause of lymphadenopathy; it occurs with increased frequency in HIV positive cases [4]. The findings of the present study were similar. Mycobacterial lymphadenitis was the most common lesion observed in HIV positive patients in the present study, which was attributed to the decreased immunological ability of the body to get rid of the mycobacterium in these patients [10]. In contrary to this result, the most common causes in HIV negative patients were reactive hyperplasia, which is constant in developed countries; reactive hyperplasia is a more common cause than infection or lymphoma [10]. Our results also indicated that the cause of cervical lymphadenopathy is different between HIV/AIDS patients and HIV-negative patients. The positive rate of Mycobacterial infection that are mainly tuberculosis in HIV/AIDS patients is significantly higher and the positive rate of Epidermal inclusion cyst and Metastatic carcinoma is markly lower than that in HIV negative patients. The results would help to increase the awareness of physicians When encountering different patients to make right diagnoses and implement empirical treatment as quickly as possible, especially in resource limited regions in China.

With the advent of HIV, mycobacterial infection and tuberculosis (TB) have turned into a primary cause of morbidity and mortality [11]. HIV infection is the critical risk factor that favors progression to active tuberculosis from latent infection by suppressing the immune response against tuberculosis [12]. It is reported that 25–65% of HIV-infected persons develop active TB of one organ or the other in their lifetime [13]. In the present study, TB accounted for 34.2% of the cases in HIV positive patients. TB infection points to a systemic disease, and with rising degrees of immunodeficiency, extrapulmonary TB is more frequent. More recent publications suggest that TB lymphadenitis is the most common form of extrapulmonary TB [14, 15]. Cervical group of lymph nodes showed a higher incidence of tuberculosis than the other sites [16].

FNAC was found to act as an approach with quite precise sensitivity and specificity in diagnosing TB [4]. Studies have shown that FNAC was more sensitive for the diagnosis of TB in HIV positive cases than in HIV negative cases [17]. The most common cytomorphological category of tuberculous lymphadenitis in the present study was caseous necrosis with epithelioid cell granuloma and multinucleated giant cells. Necrotizing suppurative inflammation and neutrophilic aggregates were also observed, which is consistent with some studies [6, 7, 18].

Acid-fast staining of tuberculosis confirmed the diagnosis, and its positivity ranged from 20.8 to 97.2% [9, 19]. Acid-fast staining was positive in 78.7% of the AIDS patients and 47.1% of the non-AIDS patients in the study, thus demonstrating a noticeably larger rate of positivity in AIDS patients than in the HIV negative cases; this result was similar to another study that demonstrated a higher acid-fast staining positivity in HIV positive patients and untreated patients [20]. The diagnostic role of FNAC and acid-fast staining has also been reported and it was found to be more reliable in patients with HIV infection because of the higher mycobacterial burden and should be the first line of investigation in these patients [12]. However, negative acid-fast staining on FNAC did not rule out tuberculosis. If the morphological findings of cytologic smears are observed to be suggestive of TB, further investigations should be advised.

Fungal opportunistic infections were one of the most frequent causes of cervical lymphadenopathy in HIV/AIDS patients, including cryptococcosis (1.5%), Talaromyces marneffei (1.5%), and other fungi (0.4%).With different climatic and social-economic conditions, the types and frequencies of opportunistic infections change from region to region [21]. Talaromyces marneffei was reported to be more common in Southern and Southwestern China, where the humid climate and ecological conditions were almost the same as that of Southeast Asia [22].

The diagnosis of metastatic carcinoma of the lymph node on a cytological smear is vital and feasible, most of which can be considered by their cytomorphological features. The primary finding is the presence of foreign cell bodies with a reactive population of lymphocytic background. A cytological diagnosis will help avoid further surgery required to confirm metastasis. FNAC not only confirms metastatic tumor cells but also gives a clue regarding the site of the primary combing cell block and IHC. Most of the studied metastatic carcinoma in head and neck were metastatic squamous cell carcinomas in HIV negative patients, accompanied by metastatic adenocarcinomas. Other studies have also found similar findings [23]. Only three cases with metastatic squamous cell carcinoma were reported in HIV positive patients, thus confirming that the incidence of metastatic carcinoma was significantly lower in HIV positive patients. This may be related to the high incidence of metastasis in elder patients with cancer. The average age of onset of metastatic carcinoma in HIV negative patients in this study was significantly higher than that of HIV positive patients.

FNAC refers to a significant diagnostic method for lymphomas since it has the ability to differentiate lymphomas from the carcinomas. Cytomorphology of FNAC and immunohistochemical studies of cell blocks have been reported to offer very high accuracy in the diagnosis of lymphoma and allowed further sub-classification in many cases [24]. In the present study, lymphomas affected 2.6% of the HIV/AIDS patients and 6.6% of the HIV negative patients. The incidence of lymphoma in HIV/AIDS patients was significantly lower than that in HIV negative patients. This may be attributed to the fact that only cervical lymph nodes were evaluated. Literature suggests that the frequency of lymphomas ranges between 5 and 41% [25]. Low frequencies of lymphomas were observed in Asian and African studies, probably because of the high frequency of infectious diseases, primarily tuberculosis, in those countries [26].

Notably, the negative cytologic results do not exclude malignancy, in particular, lymphoma. Undesirable aspirates found in different studies range from 6 to 15% [27], higher than that identified in the present study (0.7%). Undesirable aspirates were due to insufficiently trained cytopathologists and poor handling of the aspirated material [28]. It has been reported that FNAC has more diagnostic accuracy when performed by an experienced pathologist, in comparison to the other clinicians [1]. This finding is in accordance with other studies that have shown that the success of FNAC highly depends on the training and experience of the personnel conducting the procedure [28, 29]. An experienced pathologist can assess adequacy repeatedly on-site until adequate material is obtained [26].

## Conclusions

In conclusion, there were significantly different reasons for cervical lymphadenopathy in HIV-infected and non-HIV infected patients. FNAC was found to be a useful diagnostic method for differential diagnosis.

## Data Availability

The datasets used and/or analyzed during the current study available from the corresponding author on reasonable request.

## Abbreviations

FNAC: Fine needle aspiration cytology
AIDS: Acquired Immunodeficiency Syndrome
IHC: Immunohistochemical
HE: Haematoxylin and eosin
PAS: Periodic Acid Schiff
GMS: Gomori’s methenamine silver
TB: Tuberculosis
MAC: *Mycobacterium avium* complex;

## References

[1] Khan S, Liomba G, Rosenberg NE, Stanley C, Kampani C, Dhungel BM (2018). Utilization of fine needle aspiration cytology at Kamuzu central hospital. PLoS One.

[2] Nasser SS, Patil RK, Kittur SK (2017). Cytomorphological analysis of lymph node lesions in HIV-positive patients with CD4 count correlation: a cross-sectional study. Acta Cytol.

[3] Batni G, Gaur S, Sinha ON, Agrawal SP, Srivasatva A (2016). A clinico-pathological study of cervical lymph nodes. Indian J Otolaryngol Head Neck Surg.

[4] Muyanja D, Kalyesubula R, Namukwaya E, Othieno E, Mayanja-Kizza H (2015). Diagnostic accuracy of fine needle aspiration cytology in providing a diagnosis of cervical lymphadenopathy among HIV-infected patients. Afr Health Sci.

[5] Austin RM, Birdsong GG, Sidawy MK, Kaminsky DB (2005). Fine needle aspiration is a feasible and accurate technique in the diagnosis of lymphoma. J Clin Oncol.

[6] Gosavi AV, Sulhyan KR, Shetty DS, Murarkar PS, Jadhav RM (2017). FNAC of lymph nodes in HIV positive patients-a diagnostic boon. J Am Soc Cytopathol.

[7] Tirumalasetti N, Prema LP (2014). Lymph nodes cytology in HIV seropositive cases with haematological alterations. Indian J Med Res.

[8] Vanisri HR, Nandini NM, Sunila R (2008). Fine-needle aspiration cytology findings in human immunodeficiency virus lymphadenopathy. Indian J Pathol Microbiol.

[9] Shenoy R, Kapadi SN, Pai KP, Kini H, Mallya S, Khadilkar UN, Prabha A (2002). Fine needle aspiration diagnosis in HIV related lymphadenopathy in Mangalore. India Acta Cytol.

[10] Lang UT, Khalbuss EW, Monaco ES, Michelow P, Pantanowitz L (2011). Review of HIV-related cytopathology. Pathol R Int.

[11] Chand P, Dogra R, Chauhan N, Gupta R, Khare P (2014). Cytopathological pattern of tubercular lymphadenopathy on FNAC: analysis of 550 consecutive cases. J Clin Diagn Res.

[12] Jaryal A, Raina R, Sarkar M, Sharma A (2011). Manifestations of tuberculosis in HIV/AIDS patients and its relationship with CD4 count. Lung India.

[13] Sharma SK, Mohan A, Kadhiravan T (2005). HIV-TB co-infection: epidemiology, diagnosis and management. Indian J Med Res.

[14] Handa U, Mundi I, Mohan S (2012). Nodal tuberculosis revisited: a review. J Infect Dev Ctries.

[15] Fontanilla JM, Barnes A, von Reyn CF (2011). Current diagnosis and Management of Peripheral Tuberculous Lymphadenitis. Clin Infect Dis.

[16] Canberk S, Longatto-Filho A, Schmitt F (2016). Molecular diagnosis of infectious diseases using cytological specimens. Diagn Cytopathol.

[17] Shriner KA, Mathisen GE, Goetz MB (1992). Comparison of mycobacterial lymphadenitis among persons infected with human immunodeficiency virus and seronegative controls. Clin Infect Dis.

[18] Deshmukh AT, Jagtap MW, Nafees N (2013). Cytological evaluation of lymphadenopathy in HIV patients. Int J Recent Trends Sci Technol.

[19] Kumar N, Gupta BB, Sharma B, Kaushal M, Rewari BB, Sundriyal D (2015). Role of fine-needle aspiration cytology in human immunodeficiency virus–associated lymphadenopathy: a cross-sectional study from northern India. Hong Kong Med J.

[20] Sinha S, Chatterjee M, Bhattacharya S, Pathak SK, Mitra RB, Karak K, Mukherjee M (2003). Diagnostic evaluation of extra-pulmonary tuberculosis by fine needle aspiration (FNA) supplemented with AFB smear and cultures. J Indian Med Assoc.

[21] Xiao J, Gao G, Li Y, Zhang W, Tian Y, Huang Y (2013). Spectrums of opportunistic infections and malignancies in HIV-infected patients in tertiary care hospital, China. PLoS One.

[22] Chaiwun B, Vanittanakom N, Jiviriyawat Y, Rojanasthien S, Thorner P (2011). Investigation of dogs as a reservoir of Penicillium marneffei in northern Thailand. Int J Infect Dis.

[23] Hafez NH, Tahoun NS (2011). Reliability of fine needle aspiration cytology (FNAC) as a diagnostic tool in cases of cervical lymphadenopathy. J Egypt Natl Canc Inst.

[24] Zhang S, Yu X, Zheng Y, Yang Y, Xie J, Zhou X (2014). Value of fine needle aspiration cell blocks in the diagnosis and classification of lymphoma. Int J Clin Exp Pathol.

[25] Frederiksen JK, Sharma M, Casulo C, Burack WR (2015). Systematic review of the effectiveness of fine-needle aspiration and/or core needle biopsy for subclassifying lymphoma. Arch Pathol Lab Med.

[26] Houcine Y, Romdhane E, Blel A, Ksentini M, Aloui R, Lahiani R (2018). Evaluation of fine needle aspiration cytology in the diagnosis of cervical lymph node lymphomas. J Craniomaxillofac Surg.

[27] Annam V, Kulkarni MH, Puranik RB (2009). Clinicopathologic profile of significant cervical lymphadenopathy in children aged 1-12 years. Acta Cytol.

[28] Wright CA, Pienaar JP, Marais BJ (2008). Fine needle aspiration biopsy: diagnostic utility in resource-limited settings. Ann Trop Paediatr.

[29] Etit D, Tugyan N, Avci A, Altinel D, Ayca T, Secil A (2011). An evaluation of nondiagnostic fine needle aspiration biopsy results: the importance of having an experienced cytopathologist. Turkish J Med Sci.

